# Effect of a Low-Protein Diet Supplemented with Ketoacids on Skeletal Muscle Atrophy and Autophagy in Rats with Type 2 Diabetic Nephropathy

**DOI:** 10.1371/journal.pone.0081464

**Published:** 2013-11-26

**Authors:** Juan Huang, Jialin Wang, Lijie Gu, Jinfang Bao, Jun Yin, Zhihuan Tang, Ling Wang, Weijie Yuan

**Affiliations:** Department of Nephrology, Shanghai Jiaotong University Affiliated First People's Hospital, Shanghai, China; National Institute of Agronomic Research, France

## Abstract

A low-protein diet supplemented with ketoacids maintains nutritional status in patients with diabetic nephropathy. The activation of autophagy has been shown in the skeletal muscle of diabetic and uremic rats. This study aimed to determine whether a low-protein diet supplemented with ketoacids improves muscle atrophy and decreases the increased autophagy observed in rats with type 2 diabetic nephropathy. In this study, 24-week-old Goto-Kakizaki male rats were randomly divided into groups that received either a normal protein diet (NPD group), a low-protein diet (LPD group) or a low-protein diet supplemented with ketoacids (LPD+KA group) for 24 weeks. Age- and weight-matched Wistar rats served as control animals and received a normal protein diet (control group). We found that protein restriction attenuated proteinuria and decreased blood urea nitrogen and serum creatinine levels. Compared with the NPD and LPD groups, the LPD+KA group showed a delay in body weight loss, an attenuation in soleus muscle mass loss and a decrease of the mean cross-sectional area of soleus muscle fibers. The mRNA and protein expression of autophagy-related genes, such as Beclin-1, LC3B, Bnip3, p62 and Cathepsin L, were increased in the soleus muscle of GK rats fed with NPD compared to Wistar rats. Importantly, LPD resulted in a slight reduction in the expression of autophagy-related genes; however, these differences were not statistically significant. In addition, LPD+KA abolished the upregulation of autophagy-related gene expression. Furthermore, the activation of autophagy in the NPD and LPD groups was confirmed by the appearance of autophagosomes or autolysosomes using electron microscopy, when compared with the Control and LPD+KA groups. Our results showed that LPD+KA abolished the activation of autophagy in skeletal muscle and decreased muscle loss in rats with type 2 diabetic nephropathy.

## Introduction

Type 2 diabetic nephropathy is the most common cause of end-stage renal disease (ESRD). Thus, the treatment strategies for renal failure, including the use of protein-restricted diets [1], have gained increased interest for the treatment of patients with diabetic nephropathy. Despite the few studies that suggest that protein intake restriction fails to improve renal prognosis in type 1 or type 2 diabetic patients with incipient or overt nephropathy[2] and confers renoprotection[3], several findings have demonstrated that a low-protein diet preserves renal function and structure in animal models [4] and in type 2 diabetic patients with macroalbuminuria[5] and improves disease prognosis[6], low-grade inflammation and proteinuria[7] and depressive symptoms[8]. However, there has been increasing concern regarding the risk of the subsequent development of malnutrition due to these diets. Ketoacid, a nitrogen-free ketoanalog, has been prescribed together with a low-protein diet in patients with CKD [9], [10]. Ketoacids provide a sufficient amount of essential amino acids and reduce endogenous urea formation, toxic ions and metabolic products. Animal studies have shown that LPD slows down growth and decreases serum albumin levels, and supplementation with ketoacids may correct these abnormalities in rats with CKD [11]. In addition, results of a clinical trial have suggested that protein restriction supplemented with essential amino acids and ketoanalogs delays the onset of end-stage renal failure without deteriorating the nutritional status of patients with nondiabetic [12], [13] or diabetic nephropathy [14], [15]. However, the mechanism underlying this phenomenon is still unclear.

Muscle atrophy, a manifestation of malnutrition and wasting, occurs in patients with chronic kidney disease, diabetes and other conditions or diseases, such as denervation, sepsis, and cardiac failure. It has been well documented that the ubiquitin-proteasome system (UPS) contributes to muscle atrophy[16]. Moreover, the autophagy-lysosome pathway (ALP) has also emerged as an important protein degradation pathway involved in muscle atrophy. Upon induction, the isolation membrane elongates and subsequently encloses a portion of protein and dysfunctional organelles, which results in the formation of a double-membrane structure, the autophagosome. Then, the outer membrane of the autophagosome fuses with a lysosome to form an autolysosome, where their content is digested by lysosomal hydrolases. Recent studies have demonstrated autophagy activation in skeletal muscle in a variety of conditions and diseases, such as denervation and fasting [17]. Furthermore, Lecker [18] reported that the mRNA expression of autophagy-related genes (ATGs) LC3, Gabarapl1 and Cathepsin L are upregulated in the skeletal muscle of rats with streptozotocin-induced diabetes mellitus and uremia induced by a subtotal nephrectomy, demonstrating autophagy activation in the skeletal muscle of diabetic and uremic rats. However, further studies are required to confirm autophagy activation in rats with diabetic nephropathy.

Goto-Kakizaki rats exhibit a spontaneous polygenic form of diabetes and have been widely established as a genetically determined rodent model of human type 2 diabetes[19]. This rat model is generated using normal Wistar rats that have undergone repetitive selective breeding and represents a spontaneous non-insulin-dependent diabetes animal model[20]. In this study, we investigated the effects of LPD+KA on muscle atrophy in 24-week-old Goto-Kakizaki rats with spontaneous type 2 diabetes-induced nephropathy. Measurements of the blood urea nitrogen (BUN), urinary protein and serum creatinine (Scr) levels confirmed the onset of diabetic nephropathy. First, we tested the effect of a low-protein diet supplemented with ketoacids on muscle atrophy in 24-week-old GK rats. Second, given the importance of autophagy in skeletal muscle atrophy, we examined the rat muscles for abnormalities in autophagy and evaluated the effect of a low-protein diet supplemented with ketoacids on autophagy in skeletal muscle. Taken together, these results support a mechanism in which a low-protein diet supplemented with ketoacids may attenuate muscle loss in patients with diabetic nephropathy.

## Methods

### Animals and experimental design

All of the experiments were approved by the Animal Care and Use Committee of Shanghai Jiao Tong University and were performed in accordance with the National Institute of Health Guide for the Care and Use of Laboratory Animals. Male GK rats and male Wistar rats, aged 20 weeks and weighing 350±10 g, were purchased from the Chinese Academy of Sciences, Shanghai, China. The rats were individually housed for 4 weeks with a standard diet before they were fed with specific diets. At the age of 24 weeks, the onset of diabetic nephropathy in the GK rats was confirmed. The levels of serum creatinine (Scr), blood urea nitrogen (BUN) and 24 h urinary proteins were significantly enhanced in GK rats compared to Wistar rats. GK rats were randomly assigned to three dietary groups: a normal protein diet (22% protein, NPD, n = 7), a low-protein diet (6% protein, LPD, n = 7), or a low-protein diet supplemented with ketoacids (5% protein and 1% ketoacids, LPD + KA, n = 7). Ketoacids (compound α-ketoacid) were provided by Fresenius-Kabi (Beijing, China). In all three animal groups, the GK rats were paired on the basis of their body weight and blood glucose, serum albumin, Scr and BUN levels. In the control group, Wistar rats were fed with a normal protein diet (22% protein, n = 7). All three diets were modified from AIN-93 (American Institute of Nutrition Rodent Diets) and contained the same calorie content (3.5 kcal/g) and the same vitamins and minerals. The animals were maintained in cages at 22°C under a 12-h light/12-h dark cycle and were allowed free access to water. Body weights were measured every 8 weeks. In addition, urinary protein concentrations were determined by placing the animals in individual metabolic cages for a timed urine collection every 8 weeks. Blood and soleus muscle samples were collected after the animals were sacrificed. In addition, the serum albumin, Scr, blood glucose and BUN levels were also measured. Muscle samples were cleaned of any visible connective and adipose tissues and blood, and then weighed. Next, the samples were divided into three sample groups for either electron microscopy, HE staining, or the samples were immediately frozen and kept at −80°C for real-time PCR and western blotting analyses.

### Histological Analysis

The soleus muscle was immediately embedded in OCT (Tissue-Tek, USA) after the biopsies and sectioned using a cryostat (Leica CM 1900, Germany). Images were obtained using an Olympus BX51 microscope equipped with an Olympus DP73 digital camera and cellSens Digital image analysis software after HE staining. The myofiber sizes were measured using ImageJ software (National Institutes of Health, USA), and the muscle fiber CSA was calculated via the analysis of 50 myofibers of a muscle from each rat. The fiber CSA was determined from five areas at 200× magnification.

Transmission electron microscopy (TEM) using soleus muscle tissue was performed for the visualization and quantitation of autophagic structures. Muscle samples were fixed in 2.5% glutaraldehyde in 0.1 M cacodylate buffer. The specimens were post-fixed in 1% osmium tetroxide in the same buffer, dehydrated with a graded series of ethanol and embedded in Epon 812 resin. Ultra-thin sections were prepared, stained with uranyl acetate and lead citrate. TEM were obtained using a Hitachi H-7650 transmission electron microscope. For quantitative analysis, 50 random fields per animal were taken and examined at×40000. Autophagosomes were identified when meeting at least two of the following criteria: double membrane, absence of ribosome in cytosolic side of the vacuole, similar density of the luminal side of the vesicle compared with cytosol, complete or remains of organelles inside the vesicle. Single-membrane vesicles containing dense or clear amorphous material were considered as autolysosomes. The term autophagic vesicle refers to autophagosome or autolysosome[21].

### Real-time PCR

Total RNA was extracted from tissues using TRIzol reagent (15596-026, Gibco). Reverse transcription was performed using a High Capacity cDNA Reverse Transcription Kit (Thermo, #K1622) with random oligo-dT priming according to the manufacturer's protocols. PCR was performed using an ABI PRISM Sequence Detector System 7300 (Applied Biosystems, USA) with SYBR Green (F-415XL, Thermo). The PCR primers used were: rat LC3B, forward: 5′-TTTGTAAGGGCGGTTCTG-3′, and reverse: 5′-GAAGTGGCTGTATGTCTGTC-3′; rat Beclin1, forward: 5′-ACCGACTTGTTCCCTATG-3′, and reverse: 5′-CCTCCAGTGTCTTCAATC-3′; rat Bnip3, forward: 5′-GCTCCCAGACACCACAAGAT-3′ and reverse: 5′-GCT ACA ATA GGC ATC AGT CTG ACA-3′; p62, forward: 5′-GCTATTACAGCCAGAGTCAAGG-3′, and reverse: 5′-TGGTCCCATTCCAGTCATC-3′; rat Cathepsin L, forward: 5′-AGGGAGTGAGGCAGATAGG-3′, and reverse: 5′- TAGAAGGGAGCCAGGTAGG-3′. GAPDH, forward: 5′-GTCGGTGTGAACGGATTTG-3′, and reverse: 5′-TCCCATTCTCAGCCTTGAC-3′ was used as an endogenous loading control.

### Western blotting and antibodies

Tissue lysates were prepared from samples frozen in liquid nitrogen. The samples were pulverized and lysed in RIPA buffer for 2 hours at 4°C. Lysates were centrifuged at 10,000 *g* for 10 minutes at 4°C, and the supernatants were transferred into separate tubes. Equal volumes (20 µg) of protein were separated using SDS-PAGE and transferred onto nitrocellulose membranes. The membranes were incubated overnight at 4°C in 5% skim milk with primary antibodies. The following antibodies were used: GAPDH (2251-1, Fermentas, Canada), LC3B (ab63817, Abcam, UK), Beclin-1: (ab62557, Abcam, UK), Bnip3: (3485-1, Epitomics, USA), Cathepsin L: (5886-1, Epitomics, USA), p62: (ab56416, Abcam, UK). Next, the membranes were washed and incubated using a secondary anti-rabbit IgG (A0208, Beyotime Institute of Biotechnology, Shanghai, China) antibody or anti-mouse IgG (A0216, Beyotime Institute of Biotechnology, Shanghai, China) antibody conjugated with horseradish peroxidase (Beyotime Institute of Biotechnology, Shanghai, China). Band visualization was performed using an ECL Western Blotting Substrate kit (WBKLS0100, Millipore, USA).

### Statistical analysis

Values are expressed as the mean ± SD. Differences were determined using analysis of variance (ANOVA) followed by the Student-Newman-Keuls test. P-values less than 0.05 were considered statistically significant.

## Results

### Urinary protein and biochemical parameters

**Figure 1:**
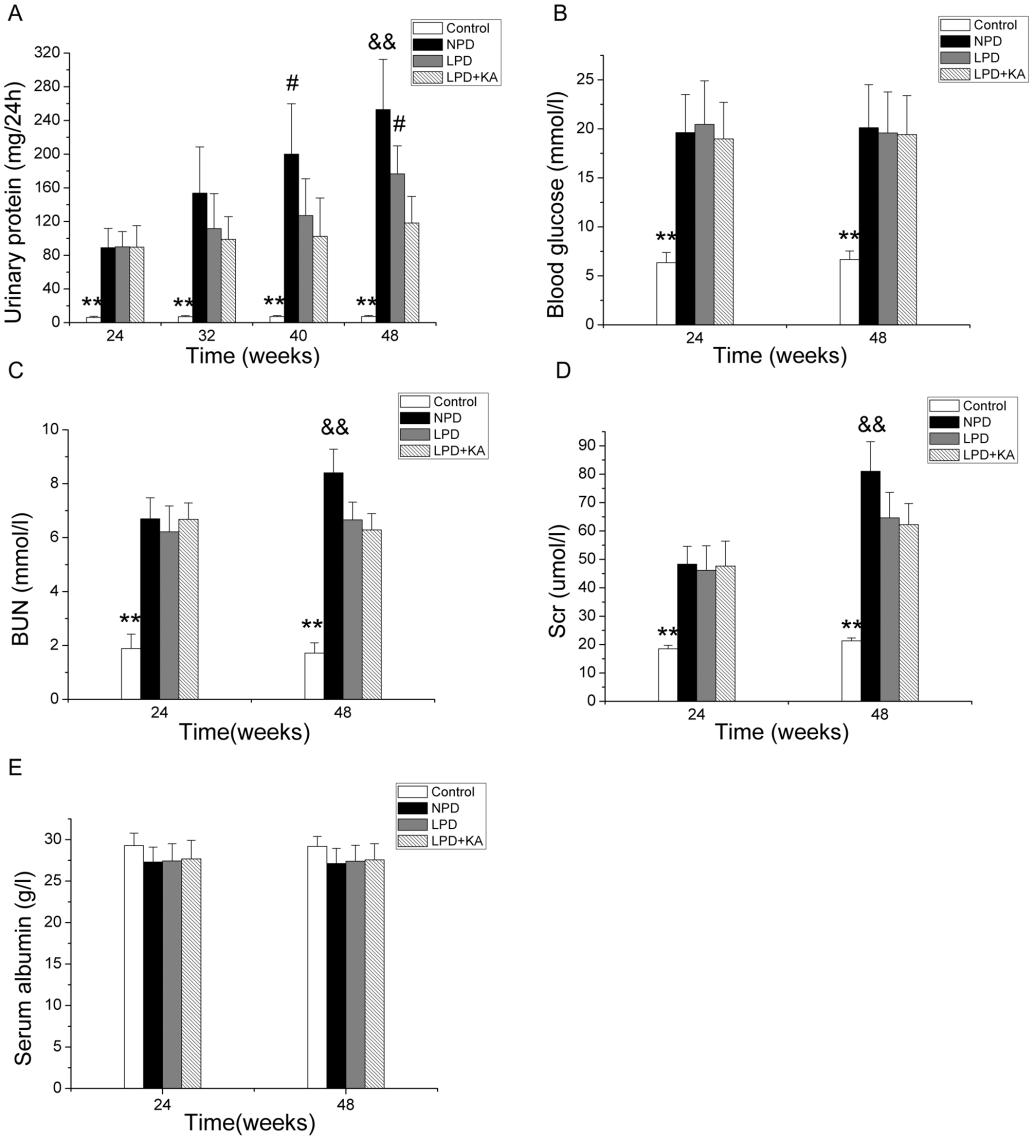
Urinary protein and biochemical parameters in the experimental groups. Urinary protein (A), blood glucose (B), BUN(C), Scr (D) and serum albumin levels (E) in Wistar and GK rats fed with NPD, LPD and LPD+KA. Data are expressed as the mean ± SD, ^**^p<0.01 versus NPD, LPD and LPD+ KA; ^#^p<0.05 versus LPD+ KA; ^&&^p<0.01 versus LPD and LPD+ KA. BUN, blood urea nitrogen; Scr, serum creatinine; NPD, normal protein diet; LPD, low-protein diet; LPD+KA, low-protein diet supplemented with ketoacids.

The urinary protein levels were remarkably higher in the GK rats compared to the Wistar rats from 24 weeks to 48 weeks (Figure 1A). At 32 weeks, there was no or very little effect of the dietary intervention; however, at 40 weeks of age, a low-protein diet supplemented with ketoacids reduced the levels of urinary protein compared to the NPD group. Furthermore, at 48 weeks, a significant difference was observed between the LPD+KA and LPD groups. Blood glucose values also increased in the GK rats, although there was no effect of the dietary intervention (Figure 1B). The BUN and Scr levels, which were higher in the GK rats, were significantly reduced with protein restriction (Figure 1C and 1D). However, unlike the parameters previously mentioned, there was no difference in the serum albumin levels between the GK and Wistar rats both at 24 weeks and 48 weeks of age, although there was a trend for a reduction in levels in GK rats (Figure 1E).

### Body weight and soleus muscle mass

**Figure 2:**
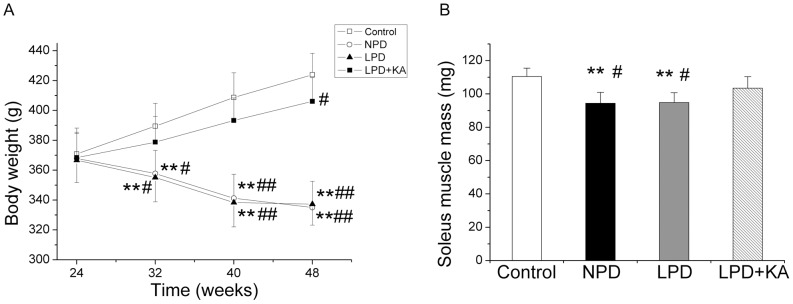
Body weight loss and soleus muscle mass loss in the experimental groups. Means ± SEM of Body weight (A) and soleus muscle mass (B) of Wistar rats and GK rats fed with NPD, LPD and LPD+KA. Data are expressed as the mean ± SD, ^**^p<0.01 versus Control group; ^#^p<0.05 versus LPD+KA; ^##^p<0.01 versus LPD+KA. NPD, normal protein diet; LPD, low-protein diet; LPD+KA, low-protein diet supplemented with ketoacids; NPD, normal protein diet.

In Wistar rats, body weight progressively increased from 24 to 48 weeks old. In contrast, the body weights decreased in GK rats fed with NPD and LPD. There was no difference between the rats fed with NPD and the rats fed with LPD. However, the loss in body weight was partially corrected with KA supplementation because body weight had progressively increased in the LPD+KA group. Although the body weight was lower in the LPD+KA group compared to the control group at 48 weeks, no significant difference was observed at 32 weeks and 40 weeks (Figure 2A). Moreover, the soleus muscle mass of GK rats fed with NPD and LPD and sacrificed at 48 weeks was lower compared to that of the Wistar rats. Furthermore, the patterns of body weight changes in the GK rats were nearly the same in the LPD and NPD groups. In addition, the low protein diet supplemented with ketoacids partially decreased the soleus muscle mass loss compared to the NPD and LPD groups (Figure 2B).

### Histological Analysis

**Figure 3:**
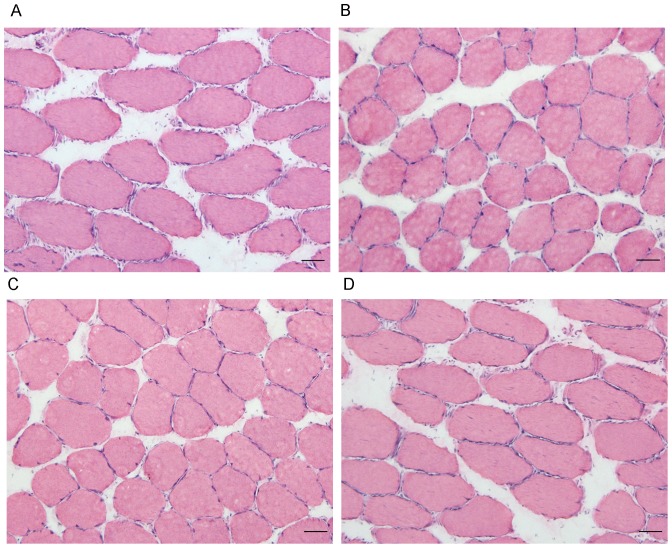
HE staining of the soleus muscle in the experimental groups. Representative HE stain images the control group (A), NPD group (B), LPD group (C) and LPD+KA group (D). NPD, normal protein diet; LPD, low-protein diet; LPD+KA, low-protein diet supplemented with ketoacids.

**Figure 4:**
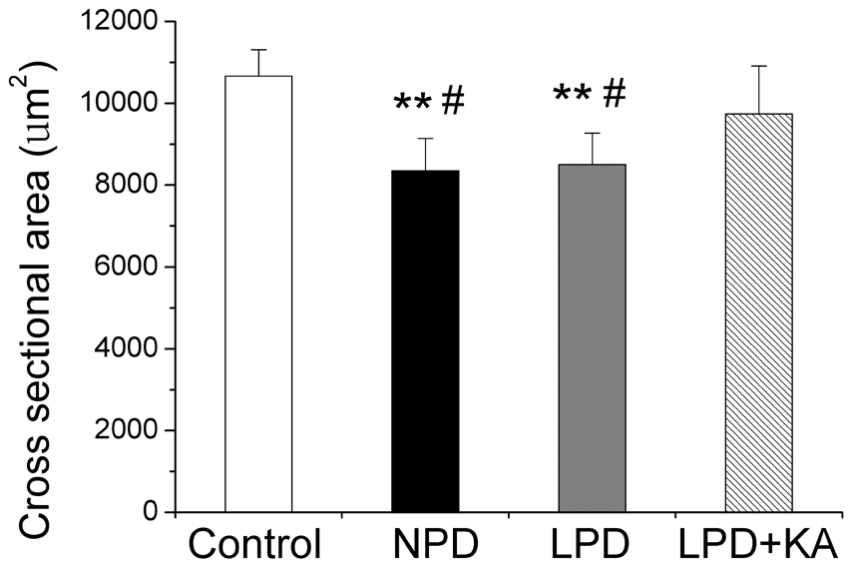
Mean cross-sectional area of soleus muscle fibers in the experimental groups. Data are expressed as the mean ± SD, scan bars: 50 µm. **p<0.01 versus Control; ^#^p<0.05 versus LPD+KA. NPD, normal protein diet; LPD, low-protein diet; LPD+KA, low-protein diet supplemented with ketoacids.

The mean CSA of the soleus muscle fibers was reduced in the NPD and LPD groups by 21.7% and 20.3%, respectively, compared to the control group. However, ketoacid supplementation increased the mean CSA of soleus muscle fibers (Figure 3 and Figure 4).

### Autophagy

**Figure 5:**
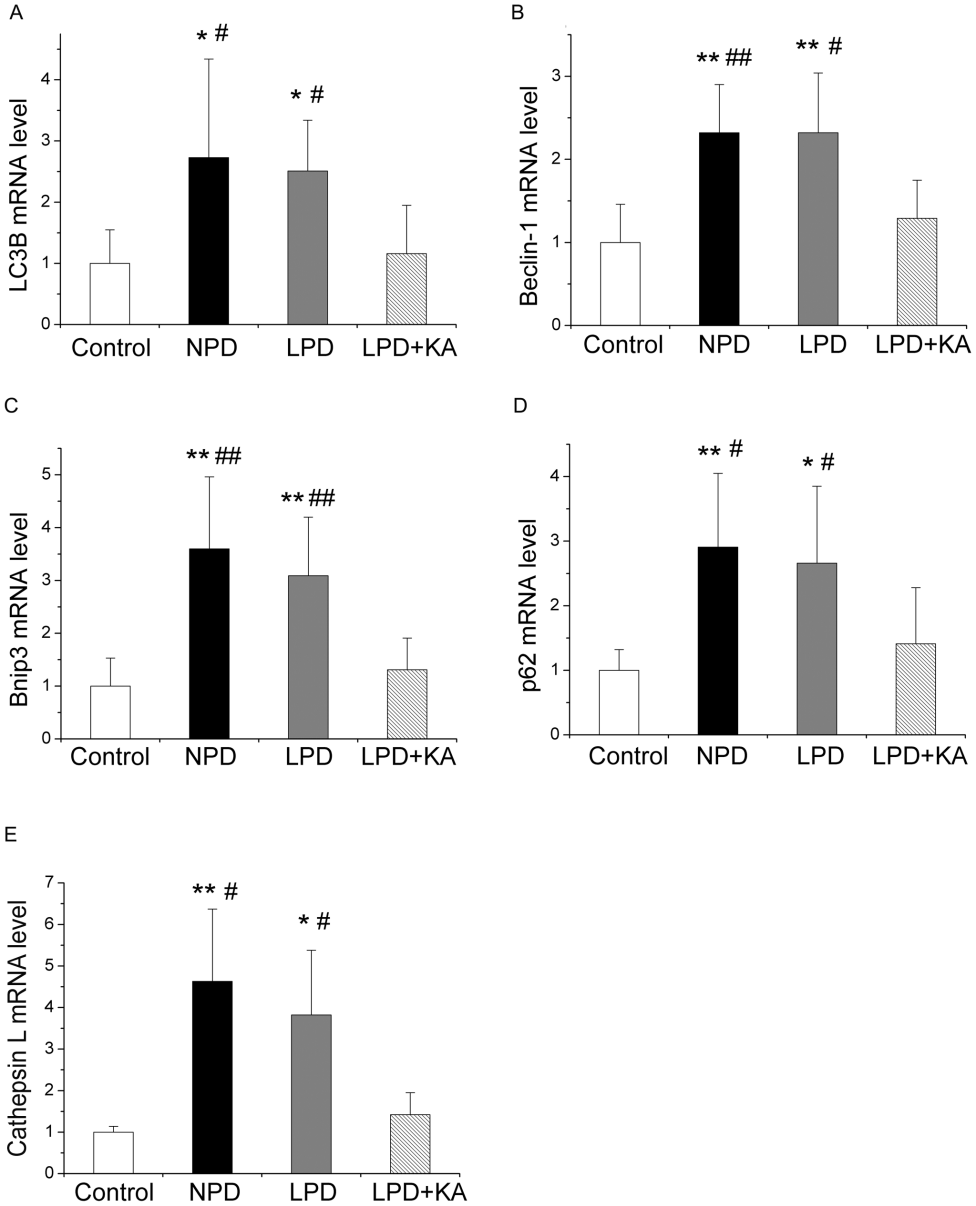
mRNA expressions of the autophagy markers in the experimental groups. LC3B (A), Beclin-1 (B), Bnip3 (C), p62 (D) and Cathepsin L (E) mRNA levels in the soleus muscle of the experimental groups. Data are expressed as the mean ± SD, *p<0.05 versus Control; **p<0.01 versus Control; ^#^p<0.05 versus LPD+KA; ^##^p<0.01 versus LPD+KA. NPD, normal protein diet; LPD, low-protein diet; LPD+KA, low-protein diet supplemented with ketoacids.

Compared to the Wistar rats, GK rats fed with NPD exhibited a significant induction of mRNA expression in the autophagy-related genes LC3B (Figure 5A), Beclin-1 (Figure 5B), and Bnip3 (Figure 5C). LC3B is a member of the LC3 family, a mammalian homolog of yeast Atg8, which plays a critical role in the formation of autophagosomes[22]. In addition, the Class III PI3K–Beclin1 complex plays a determinant role in the initiation of autophagy[23]. It has been reported that Bnip3, a central player in autophagy signaling, is upregulated by Foxo3, resulting in the upregulation of autophagy [17]. Furthermore, the mRNA levels of p62, which binds directly to atg8/LC3 to degrade ubiquitinated protein aggregates by autophagy [24], is higher in the soleus muscle of rats fed with NPD (Figure 5D). Taken together, these findings indicated an upregulation of autophagy, which was accompanied by an increase in lysosomal Cathepsin L mRNA levels (Figure 5E). Cathepsin L is a ubiquitously expressed lysosomal protease that mediates the removal of both organelles and non-myofibril cytosolic protein aggregates, and its upregulation indicates an upregulation of the autophagy-lysosome pathway. Moreover, a low-protein diet also caused a slight reduction in the increased expression of these parameters, although none of these changes reached statistical significance compared to treatment with NPD. As expected, ketoacid supplementation significantly reduced the upregulation of these genes. The mRNA expression levels of LC3B, Beclin-1, and Bnip3 were slightly higher in the LPD+KA group compared to the control group, although none of these changes were significantly different.

**Figure 6:**
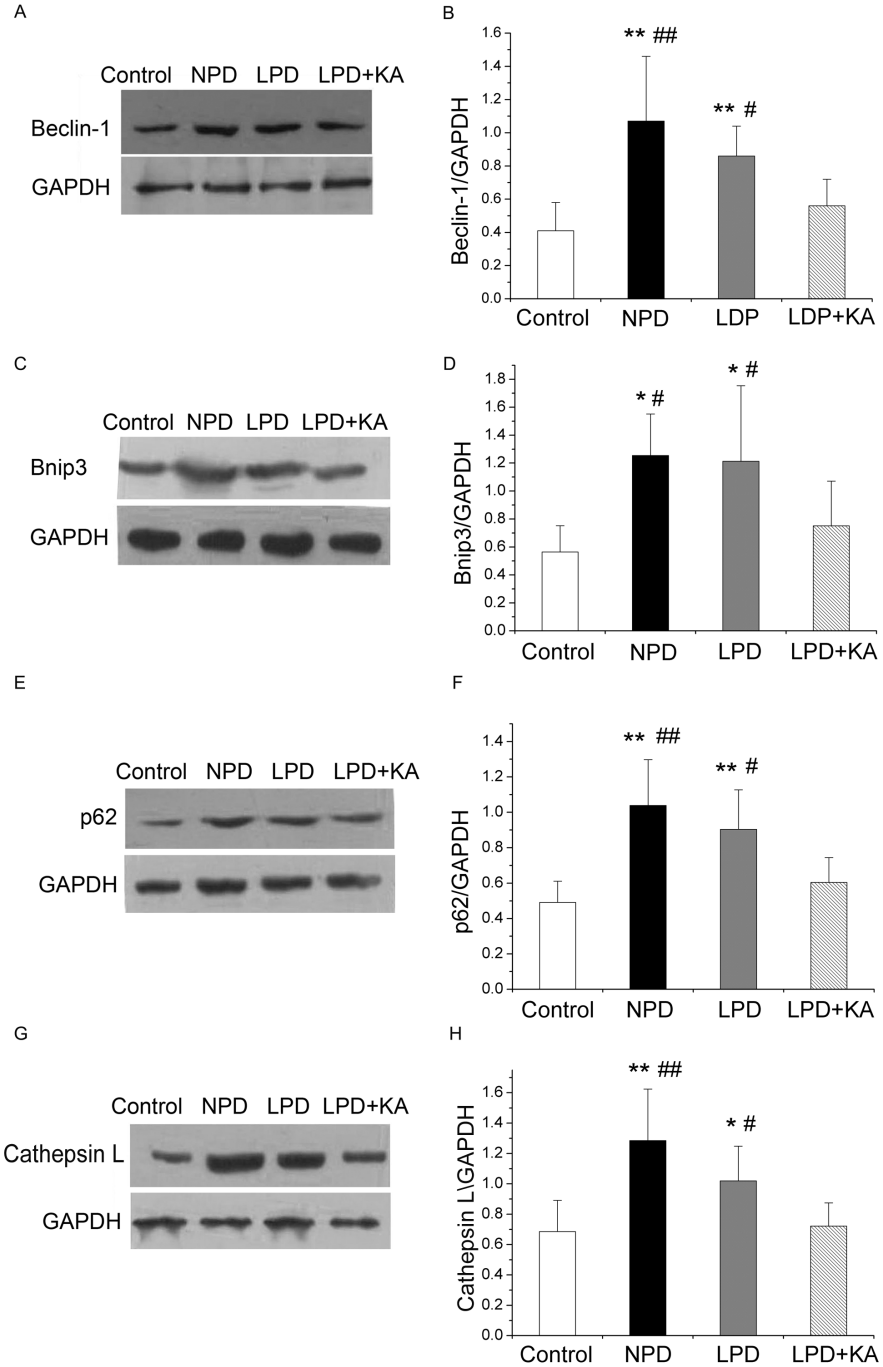
Protein expressions of the autophagy markers in the experimental groups. Representative western blotting analyses and group data of Beclin-1 (A), Bnip3 (B), p62 (C), and Cathepsin L (D) abundance in the soleus muscle of the experimental groups. Data are expressed as the mean ± SD, *p<0.05 versus Control; ^**^p<0.01 versus Control; ^#^p<0.05 versus LPD+KA; ^##^p<0.01 versus LPD+KA. LPD, NPD, normal protein diet; low-protein diet; LPD+KA, low-protein diet supplemented with ketoacids.

Similarly, GK rats fed with NPD showed a significantly higher level of protein expression in Beclin-1 (Figure 6A and 6B), Bnip3 (Figure 6C and 6D), p62 (Figure 6E and 6F) and Cathepsin L (Figure 6G and 6H) compared to Wistar rats. Moreover, a low-protein diet also caused a slight decrease in the expression of these proteins, although these changes were not statistically significant. The addition of KA to LPD prevented the overexpression of these autophagy proteins.

**Figure 7:**
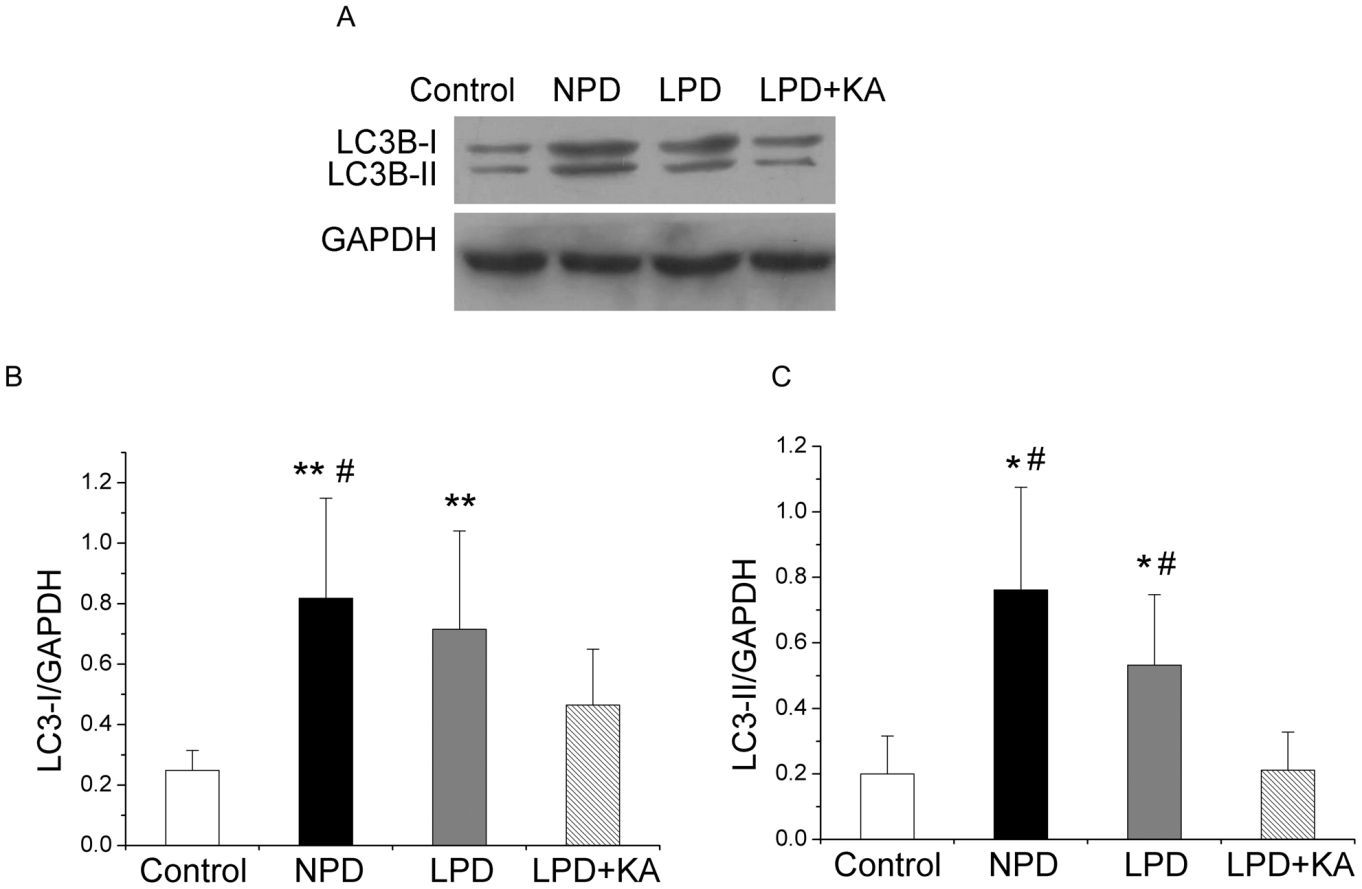
Protein expressions of LC3B in the experimental groups. Representative western blotting analyses (A) and group data of the abundance of LC3B-I (B) and LC3B-II (C) in the soleus muscle of the experimental groups. Data are expressed as the mean ± SD, *p<0.05 versus Control; ^**^p<0.01 versus Control; ^#^p<0.05 versus LPD+KA. NPD, normal protein diet; LPD, low-protein diet; LPD+KA, low-protein diet supplemented with ketoacids.

The activation of autophagy in the NPD and LPD groups was confirmed using immunoblotting for LC3B. During autophagy, soluble LC3-I is lipidated with the addition of phosphatidyl-ethanolamine into its insoluble form, LC3-II. LC3-II, which is a marker of autophagy, binds to the inner and outer membranes of the autophagosome. The level of LC3-II is correlated with the extent of autophagosome formation[22]. In the NPD and LPD groups, increased levels of membrane-bound LC3-II were observed, suggesting an increase in the conjugation of LC3 to phosphatidylethanolamine compared to the control group. Moreover, the NPD and LPD groups also exhibited an increase in LC3-I, which may reflect a feature of metal exposure that is characterized by an increase in LC3 gene expression associated with autophagy. The addition of KA to LPD also resulted in the prevention of the overexpression of LC3-II (Figure 7).

**Figure 8:**
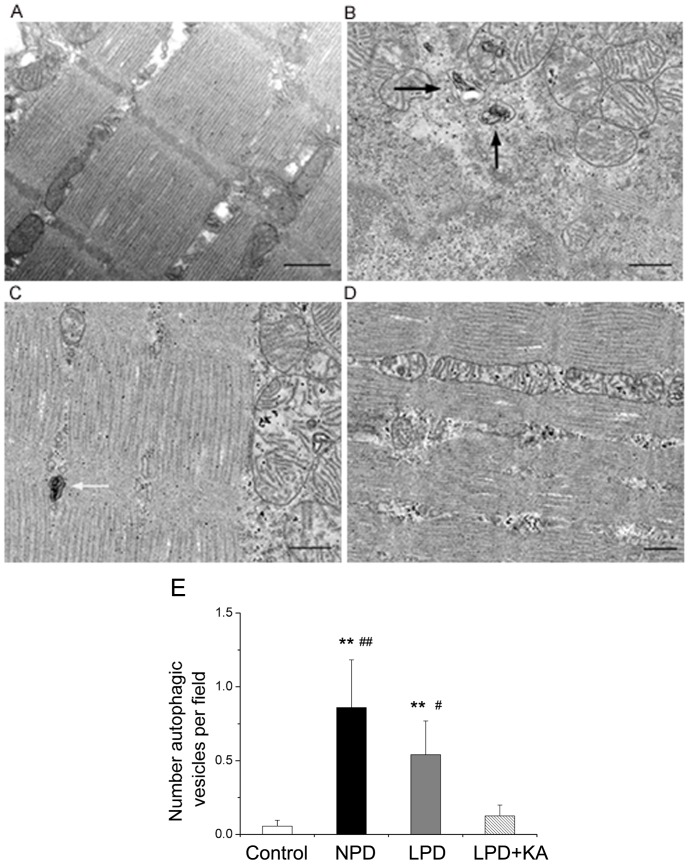
Ultrastructure of soleus muscle in the experimental groups. Representative electron micrograph of a section of the soleus muscle obtained from the control group shows normal ultrastructure and the absence of autophagosomes (A). Representative electron micrographs of a section of soleus muscle obtained from the NPD group (B) and LPD group (C) revealing autophagosomes (black arrows) or autolysosomes (white arrows). Representative electron micrograph of a section of soleus muscle obtained from the LPD+KA group demonstrating the absence of autophagosomes or autolysosomes (D). Quantification of autophagic vesicles in soleus muscles by electron micrograph (E). ^**^p<0.01 versus Control; ^#^p<0.05 versus LPD+KA; ^##^p<0.01 versus LPD+KA. NPD, normal protein diet; LPD, low-protein diet; LPD+KA, low-protein diet supplemented with ketoacids. Scale bars: 500 nm.

To further confirm that autophagy was induced in the muscles of animals in the NPD and LPD groups, muscle fiber ultrastructure was analyzed using TEM. Compared to control group (Figure 8A), NPD (Figure 8B) and LPD (Figure 8C) groups showed the formation of autophagosomes and autolysosomes. As expected, ketoacid supplementation abolished autophagy activation compared to animals fed with normal and low-protein diets, whereas few or no autophagic vesicles were observed in the muscles of animals in the LPD+KA group (Figure 8D). The quantification of these autophagic vesicles demonstrated an increase of autophagy in the muscles of mice in the NPD and LPD groups compared to control mice; mice in the LPD+KA group exhibited reduced autophagy compared to mice in the NPD and LPD groups (Figure 8E).

## Discussion

Chronic kidney disease is associated with a loss in body protein mass and fuel reserves, which is also known as protein-energy wasting (PEW). Serum albumin, a biochemical indicator that can be used in the diagnosis of PEW, is a strong predictor of mortality in maintain hemodialysis patients[25]. Furthermore, a low BMI is associated with a poor outcome and a high risk of death [26]. PEW is also associated with inflammation and cardiovascular diseases and strikingly increases mortality risk in chronic dialysis patients [27], [28]. Muscle wasting is considered one of the most valid markers of PEW in CKD and is associated with inflammation and increased mortality[29]. Muscle wasting is also common in patients with diabetes mellitus(DM) [30], and previous studies have shown that ESRD patients with diabetes mellitus are more prone to muscle wasting compared to non-diabetic ESRD patients[31], [32]. The coexistence of chronic kidney disease and DM synergistically increases muscle mass loss in ESRD patients with DM. The effects of insulin resistance/deficiency are additive to those of uremia-related factors, contributing to muscle atrophy in diabetic nephropathy. In addition, uremia per se may further substantiate the rate of increased muscle protein breakdown, primarily by worsening the level of insulin resistance. In addition to insulin resistance, there are several other potential factors that may lead to accelerated muscle protein breakdown in DM ESRD patients. For instance, diabetic patients have diabetic gastroparesis, which could lead to poor nutritional state. Thus, it is critical to identify the mechanism underlying muscle atrophy induced by diabetic nephropathy to gain insight into interventions for the improvement of nutritional status. In this study, we sought to determine the effect of a low-protein diet supplemented with ketoacids on muscle atrophy and autophagy in rats with type 2 diabetic nephropathy. In this study, we provide several novel findings. First, our data demonstrated that autophagy was upregulated in the skeletal muscles of rats with diabetic nephropathy. Second, a low-protein diet supplemented with ketoacids decreased muscle loss. Third, a low-protein diet supplemented with ketoacids decreased the increase in autophagy compared to animals on normal and low-protein diets.

For decades, protein restriction has been used to alleviate uremic symptoms, to protect the function of remnant kidneys, as well as to improve complications such as abnormal glucose metabolism and hypertension in patients with chronic kidney disease[1], [10], [33]. Furthermore, these metabolic effects resulted in less frequent prescriptions of phosphate binders, allopurinol, bicarbonate supplements[34], and erythropoietin[35]. In studies on diabetic nephropathy, several reports have demonstrated that a low-protein diet prevented the progression of renal injury in both humans and animals with diabetic nephropathy [36]. Consistent with these findings, we found that LPD decreases Scr, BUN, and urinary protein levels, although the blood glucose level was not altered. We also found that protein restriction protected against hemodynamic changes in hyperfiltration, progressive sclerosis of functional glomeruli and decreases in nitrogen waste and oxidative stress[37]. However, several reports have suggested that LPD may slightly slow the progression of renal failure, although this finding was not statistically significant [38], [39]. The effect of LPD on diabetic nephropathy has been controversial. Long-term studies examining large representative groups of patients with either type 1 or type 2 diabetic nephropathy are necessary to characterize the effect of protein restriction on diabetic nephropathy. However, the potential deleterious effects of the diet on nutritional status raises some concern [40]. Brodsky and colleagues [37] have observed that type 1 diabetic mellitus patients with early nephropathy experience protein malnutrition when eating a LPD, which may be associated with enhanced protein breakdown induced by insulin deficiency. However, Giordano has also shown that a low-protein diet is effective in reducing whole-body proteolysis and is associated with a decrease in protein oxidation and an increase in serum albumin levels in type 2 diabetic patients [7]. In this study, we found that compared with NPD, LPD did not result in a decrease in body weight. Moreover, soleus muscle mass and soleus CSA were slightly higher in GK rats fed with LPD compared to GK rats fed with NPD. However, this difference was not statistically significant, suggesting that LPD exhibits beneficial effects on renal function in the absence of deteriorating muscle wasting. With sufficient energy intake and careful monitoring for dietary compliance, a low-protein diet is nutritionally safe in patients with early type 2 diabetic nephropathy. First, a low protein diet improves appetite and physical condition, resulting in sufficient energy intake. Second, the hypercatabolic state may be suppressed because of controlled metabolic acidosis and a reduction in uremic toxins.

Nevertheless, enthusiasm regarding the use of oral nutritional supplements such as ketoacids has increased. Ketoacids capture excess nitrogen residues and utilize these residues for essential amino acid production. Thus, nitrogen intake may be restricted, and endogenous urea formation is reduced. Furthermore, if there is a sufficient amount of essential amino acids, an accumulation of non-excreted, potentially toxic ions and metabolic products arising from the breakdown of foods rich in protein may be avoided [41]. A recent animal study found that the addition of ketoacids to an LPD prevents a loss in weight gain and completely normalizes serum albumin levels, indicating that LPD supplemented with ketoacids can maintain nutritional status [11]. We found that a low-protein diet supplemented with ketoacids maintains muscle mass and decreases body weight losses, indicating that LPD supplemented with ketoacids may serve as a potential intervention that can be used to improve muscle atrophy induced by type 2 diabetic nephropathy. However, other additional potential mechanisms remain to be elucidated.

The primary role of autophagy, a highly conserved homeostatic process, is to protect cells under stressful conditions such as starvation and maintaining the amino acid pool, which is essential for survival. However, if autophagy is excessively induced, it can also result in pathological changes such as cell death and apoptosis. Recently, the activation of autophagy has been demonstrated in skeletal muscle in a variety of conditions and in disease states ranging from fasting [17], [18], oxidative stress [42], denervation [43], [44], and drug effects [45], [46] to some systemic diseases such as sepsis [47], MDC1A[48], and cancer [18]. We have reported that autophagy is activated in the muscle of rats with diabetic nephropathy using a variety of assays, including RT-PCR, which analyzed the mRNA expression of ATGs; western blotting analyses, which measured the protein levels of these ATGs; and TEM, which was used to confirm the appearance of autophagic vesicles and quantify them. Our results were consistent with a previous study that reported the upregulation of mRNA expression of LC3, Gabarapl1, and Cathepsin L in the skeletal muscle of diabetic rats and uremia rats induced by subtotal nephrectomy [18]. These findings suggest that DM in combination with uremia-related factors would act synergistically to increase autophagy in the muscles of diabetic nephropathy rats. However, while a comparative analysis of muscle mass and autophagy in a non-diabetic nephropathy model or diabetic animal without renal disease would provide the appropriate answer to the question of what the proportion each factor contributes, such an experiment was not the primary goal of this particular study and should be explored with further research. This type of additional study would allow us to better understand the mechanism by which autophagy is activated.

Importantly, Carmignac [48] reported that a systemic injection of 3-methyladenine (3-MA), an autophagy inhibitor, reduced muscle atrophy caused by MDC1A in animal models. In addition, 3-MA completely abolished lipopolysaccharide-induced muscle proteolysis [49], suggesting that the activation of ALP contributes to the development of skeletal muscle atrophy via the removal of a portion of proteins and organelles [17], [43], [50].

Given the important role of autophagy in muscle atrophy, we examined the effect of LPD and LPD+KA on autophagy in the skeletal muscle of type 2 diabetic nephropathy rats. Interestingly, we found that LPD tended to decrease the increase in autophagy, although this finding did not reach statistical significance. However, LPD+KA abolished the activation of autophagy, indicating that LPD+KA improved the muscle atrophy in diabetic nephropathy rats by abolishing the activation of autophagy.

It is well established that essential amino acids or branched-chain amino acids (BCAAs), particularly, leucine, stimulate protein synthesis in skeletal muscle. Moreover, BCAAs can activate the protein kinase mTOR, which phosphorylates 4E binding protein 1 (4E-BP1) and the 70-kDa ribosomal protein S6 kinase (S6K1), subsequently stimulating skeletal muscle protein synthesis [51], [52]. Furthermore, leucine and BCAAs decrease skeletal muscle protein degradation [53], [54]. However, a few studies have demonstrated that BCAA decreased the expression of atrogin-1, an ubiquitin-ligase enzyme, indicating that BCAAs decrease muscle protein degradation via the inhibition of the ubiquitin–proteasome system [55]. However, an animal study reported no reduction of proteasome mRNA expression or the expression of E3 atrogin-1 and muscle MuRF1; however, a significant decrease in the autophagy marker LC3-II expression in skeletal muscle was observed when rats were treated with leucine [56]. Consistent with these findings, a clinical trial showed that leucine decreased autophagy in skeletal muscle [54].

In addition, LPD is also thought to increase autophagy compared with normal protein diet due to an effect of amino acid on autophagy in skeletal muscle. Unexpectedly, LPD did not increase autophagy compared with NPD. In normal subjects, it has been suggested that metabolic adaptation results primarily from a reduction in amino acid oxidation and protein degradation induced by feeding[57]. These responses led to an increased efficiency of amino acid utilization. The protein-sparing mechanism has also been shown in patients with chronic renal failure. The amino acid oxidation values are lower in response to a lower protein intake in patients with chronic renal failure, suggesting a potential “functional reserve” for protein sparing [58], [59]. This metabolic adaptation may explain our findings. Moreover, we found a trend for LPD to decrease the induced autophagy compared to NPD. Because protein restriction exhibits many benefits, such as a slowing the rate of progression in CKD, a lessening in the accumulation of metabolic waste products, and in the improvement of insulin sensitivity [60], we surmised that these benefits contributed to the effect of LPD on autophagy in skeletal muscle. However, further studies are required to confirm this finding.

During metabolism, amino acids are deaminated or transaminated to form ketoacids via the release of an amino group. These reactions are reversible, and the use of ketoanalogs may result in the production of amino acids. Ketoacids can fully substitute for their respective essential amino acids to maintain nutritional status because these compounds maintain a neutral nitrogen balance. In this study, we observed that LPD supplemented with ketoacids abolished the activation of autophagy in the skeletal muscle compared to LPD. This finding was consistent with several previous reports on the effect of BCAAs or essential amino acids on autophagy in skeletal muscle [54], [56]. In addition, there is abundant evidence supporting the effectiveness of LPD supplemented with ketoacids compared to LPD alone in alleviating uremic symptoms[61] and in protecting the function of remnant kidneys [11], [62], [63]. We hypothesized an indirect preservation of renal function by ketoacids in decreasing autophagy in skeletal muscle. However, the mechanisms underlying the inhibition of the activation of autophagy by a low-protein diet supplemented with ketoacids remain to be investigated.

In summary, our study demonstrated that autophagy was increased in skeletal muscle in rats with diabetic nephropathy. In addition, a low-protein diet supplemented with ketoacids improved the loss in muscle mass and blocked the activation of autophagy in the skeletal muscle of rats with type 2 diabetic nephropathy. Thus, these findings may provide relevant pre-clinical data for the use of a low-protein diet supplemented with ketoacids in patients with type 2 diabetic nephropathy.
